# Special Issue: “Advances in Immunotherapy for Cancer: From Molecular Basis to Novel Biomarkers and Therapeutic Targets”

**DOI:** 10.3390/ijms26199696

**Published:** 2025-10-05

**Authors:** Laura Conti, Valentina Audrito

**Affiliations:** 1 Department of Molecular Biotechnology and Health Sciences—Molecular Biotechnology Center “Guido Tarone”, University of Turin, Piazza Nizza 44, 10126 Turin, Italy; 2 Department of Science and Technological Innovation (DISIT), University of Eastern Piedmont, Viale Teresa Michel 11, 15121 Alessandria, Italy

Over the past decade, immunotherapy has revolutionized the treatment of cancer, emerging as a novel pillar of cancer therapy and markedly improving patient survival across multiple tumor types. Yet, despite these remarkable advances, many patients are not able to benefit from immunotherapy, largely due to primary or acquired resistance mechanisms such as insufficient tumor immunogenicity, an immunosuppressive tumor microenvironment (TME), or the emergence of adaptive escape pathways. Consequently, therapy resistance and metastatic dissemination continue to pose difficult challenges that contribute to cancer-related mortality [1]. Addressing these obstacles requires a more comprehensive understanding of the molecular, metabolic, and functional interplay between malignant cells and the immune system within the TME [2,3].

This Special Issue of *IJMS* brings together recent insights into cancer immunotherapy and tumor immunology, with particular emphasis on the pivotal role of the TME in shaping tumor progression and therapeutic outcomes, as well as on the identification of novel therapeutic targets and biomarkers.

The 10 review and research articles included in this issue (see Table 1) contribute to extending our knowledge on several aspects of tumor immunology and immunotherapy, which may help with the rational development of next-generation immunotherapies and combinatorial approaches. Moreover, these advances hold promise for the discovery of predictive factors that will enable more precise monitoring of patients’ immune responses and improve personalization of treatment strategies.

Head and neck cancer (HNC) is one of the tumor types that benefited from the introduction of immunotherapy, which improved one-year survival rates in patients with recurrent metastases from 36% to 57% [4]. However, patient responses remain highly heterogeneous, highlighting the urgent need for biomarker-guided selection and the development of rational combination therapies. To deepen our understanding of HNC tumor immunology, **Arriola Benìtez PC** and colleagues reviewed the intricate interplay between the immune system and head and neck squamous cell carcinoma (HNSCC), focusing on the mechanisms of immune evasion. They discussed how HNSCC tumors frequently downregulate HLA expression, recruit immunosuppressive cells, and deregulate the expression of immune checkpoint molecules, thereby compromising therapeutic efficacy. Moreover, they provided a clear overview of the emerging interventions—including checkpoint blockade, antibody-based treatments, CAR-T cell therapy, and epigenetic modulation—and their combinations as strategies to reprogram tumor–immune cell cross-talk to improve clinical outcomes. The CAR-T approach has proven efficacy in patients with B cell malignancies, but less so in solid tumors due to limited cancer-specific targets. Emerging antigens, such as ErbB family, MUC1, CD70, and CD98hc, are subject to investigation as CAR-T cell antigens. Recently, the tight junction protein claudin 6 (CLDN6) was identified as a CAR target in solid tumors, with potential therapeutic perspectives [5].

In line with the conclusions of Arriola Benitez et al., **Mhaidly N** and co-workers analyzed the role of tumor-associated macrophages (TAMs) in HNC, with emphasis on their prognostic value and functional contribution to disease progression. To this end, the authors introduced a novel scoring algorithm, the *Macroscore*, which integrates M1 (antitumor) and M2 (protumor) macrophage ratios and densities. Interestingly, the Macroscore outperformed TNM criteria and p16 status, showing a significant association with poor patient prognosis, and demonstrated significant predictive value for overall survival. Mechanistic studies employing 3D spheroid co-culture models further revealed that tumor cells drive monocyte polarization toward an M2 phenotype, which in turn promotes tumor proliferation and epithelial to mesenchymal transition (EMT), thereby elucidating key aspects of TAM–tumor crosstalk.

Similarly, **Zaky MY** and colleagues investigated the potential of myeloid-derived infiltrates—including TAMs and myeloid-derived suppressor cells (MDSCs)—as therapeutic targets in HNSCC. Through a high-throughput screening of 70 kinase inhibitors in advanced co-culture systems, specific compounds were identified that selectively impaired the proliferation of distinct myeloid subsets. Among these, the angiogenic inhibitor of vascular endothelial growth factor receptor (VEGFR) vatalanib emerged as a promising candidate, demonstrating in vivo efficacy in reducing tumor burden and diminishing myeloid infiltration. Notably, VEGFR, despite its ubiquitous expression, is usually more upregulated in tumors compared to normal tissues, suggesting that its inhibition could be more selective in tumors [6]. These findings underscore the complexity of pharmacological modulation within the tumor myeloid compartment and position this platform as a useful tool for future therapeutic discovery in HNSCC and related malignancies.

The role of TAMs in shaping the TME and their therapeutic potential was also addressed in hepatocellular carcinoma (HCC) by **Bannister ME** et al. in their review paper. They described that TAMs exhibit a variety of functions that aid HCC tumor progression, including the promotion of angiogenesis, resistance to drug therapy, and releasing factors that support tumor cell proliferation and metastasis. However, these cells show a high plasticity that can be reprogrammed to drive tumor-specific immune responses.

Breast cancer and its interaction with the immune system are the focus of the review by **Cossu C** and collaborators, which addresses the dualistic role of Toll-like receptor 2 (TLR2) and the cGAS–STING axis in breast cancer, questioning whether these pathways are tumor-promoting or tumor-suppressive. The work aims to provide a comprehensive understanding of these pathways, with the goal of identifying potential targets for novel drug development and opening new therapeutic avenues for breast cancer. Ultimately, it stresses the importance of carefully considering the specific tumor context when modulating these receptors for therapy.

Novel therapeutic strategies for cancer treatment are reviewed by **Ruzzi F** et al., who addresses the efficacy of virus-like particles (VLPs) as both prophylactic and therapeutic vaccine platforms in oncology. Several vaccines based on VLPs have already been licensed and are commercially available, targeting HPV- and HBV-associated malignancies [7], and have recently been introduced in clinical trials as new cancer immunotherapy (an example VLPONC-01, NCT06736379), to activate targeted immune responses within the tumor microenvironment. In addition, emerging evidence highlights their therapeutic potential. Notably, preclinical and early-phase clinical investigations in HER2-positive breast carcinoma and melanoma demonstrate encouraging outcomes, supporting the hypothesis that VLPs targeting HER-2 for breast and ovalbumin or tyrosinase-related protein in melanoma may constitute a durable antitumor strategy through the activation of both innate and adaptive immune responses.

Among the novel therapeutic approaches, **Khunti N** et al. investigated the impact of CD19 monoclonal antibodies (mAbs) and peptide inhibitors of CXCR4 on antibody-dependent cell-mediated cytotoxicity (ADCC) in the context of B-cell lymphomas, with particular focus on diffuse large B-cell lymphoma (DLBCL) and the more indolent Waldenström Macroglobulinemia (WM). CD19-targeted immunotherapy has significantly improved the treatment options for various DLBCLs to WM [8]. The authors showed that interfering simultaneously with CD19 and CXCL12/CXCR4 axis could be a valid strategy to effectively inhibit CD19-mediated migration enhancement and promote ADCC, thereby augmenting the therapeutic efficacy of CD19 mAb-based immunotherapy in lymphoma models.

Immunotherapy has totally revolutionized the therapeutic standards for cancer patients. **Bloom M** and collaborators in their review paper highlighted the contribution of immune checkpoint inhibitors (ICIs) in HCC, the third leading cause of cancer-related mortality in the world. ICIs have demonstrated improved outcomes in advanced HCC and are integral to the overall treatment paradigm. However, the authors discussed the need to improve the efficacy of ICIs and described different immunotherapy combination approaches including ICIs with anti-angiogenic agents targeting VEGF or using a dual immune checkpoint blockade.

The impact of ICIs was also addressed in a melanoma model by **Trocchia M** et al. The advent of immunotherapy revolutionized the therapeutic approach to this tumor and significantly ameliorated patients’ clinical outcome. In this review, the authors specifically recapitulated the multiple roles of innate immune cells in melanoma and the related implications for immunotherapy. Indeed, growing evidence demonstrates the crucial role of innate immunity in tumor initiation, progression and response to therapy in tumors, including in cutaneous melanoma. Harnessing innate immune cells such as NK cells, macrophages, or dendritic cells, could provide complementary antitumor activity, enhancing tumor clearance and reducing the risk of immune escape. Targeting innate immunity—for example, targeting the innate immune pattern recognition receptors (PRRs)—may synergize with ICIs and adoptive T cell therapies by promoting a more inflamed tumor microenvironment and facilitating sustained adaptive immune responses [9,10].

The prediction of ICI efficacy using exome-derived variables was the focus of the article by **Dalens L** and colleagues. ICIs have improved the care of patients in multiple cancer types. However, PD-L1 status, high Tumor Mutational Burden (TMB), and mismatch repair deficiency are the only validated biomarkers of efficacy for ICIs, highlighting the clinical need to discover novel predictive biomarkers. Validated biomarkers of efficacy for ICIs remain imperfect, and new predictive markers represent an unmet medical need. Whole-exome sequencing was carried out on metastatic or locally advanced cancers from different tumor types. KRAS mutations, TMB, TCR clonality, and Shannon entropy were retained to generate an exome-derived score. This retrospective study demonstrated that exploiting this exome-derived model (exome-derived variable) combined with clinical variables allowed a better stratification of patient’s risk independently of tumor type.

**In conclusion, the research articles and reviews presented in this Special Issue collectively underscore the substantial promise of cancer immunotherapy**. By addressing current limitations and advancing innovative strategies, immunotherapy holds the potential to transform cancer into a manageable condition, slowing or preventing disease progression. Such progress could markedly reduce the global cancer burden and lead to meaningful improvements in patient outcomes.

## Figures and Tables

**Table 1:** List of the articles present in this Special Issue.

First Author	Reference
Arriola Benítez, PC	Arriola Benítez, P.C.; Fusco, M.; Amorin, R.; Picón, C.R.; Piccioni, F.; Victoria, L.; Rizzo, M.M.; Malvicini, M. Unraveling the Role of Tumor-Infiltrating Immune Cells in Head and Neck Squamous Cell Carcinoma: Implications for Antitumor Immune Responses and Immunotherapy. *Int. J. Mol. Sci.* **2025**, *26*, 6337. https://doi.org/10.3390/ijms26136337
Mhaidly, N	Mhaidly, N.; Journe, F.; Najem, A.; Stock, L.; Trelcat, A.; Dequanter, D.; Saussez, S.; Descamps, G. Macrophage Profiling in Head and Neck Cancer to Improve Patient Prognosis and Assessment of Cancer Cell–Macrophage Interactions Using Three-Dimensional Coculture Models. *Int. J. Mol. Sci.* **2023**, *24*, 12813. https://doi.org/10.3390/ijms241612813
Zaky, MY	Zaky, M.Y.; John, J.; Vashisht, M.; Singh, P.; Al-Hatamleh, M.A.I.; Siddoway, K.; Chen, Z.; Wang, J.H. Targeting Myeloid Cells in Head and Neck Squamous Cell Carcinoma: A Kinase Inhibitor Library Screening Approach. *Int. J. Mol. Sci.* **2024**, *25*, 12277. https://doi.org/10.3390/ijms252212277
Bannister, ME	Bannister, M.E.; Chatterjee, D.A.; Shetty, S.; Patten, D.A. The Role of Macrophages in Hepatocellular Carcinoma and Their Therapeutic Potential. *Int. J. Mol. Sci.* **2024**, *25*, 13167. https://doi.org/10.3390/ijms252313167
Cossu, C	Cossu, C.; Di Lorenzo, A.; Fiorilla, I.; Todesco, A.M.; Audrito, V.; Conti, L. The Role of the Toll-like Receptor 2 and the cGAS-STING Pathways in Breast Cancer: Friends or Foes? *Int. J. Mol. Sci.* **2024**, *25*, 456. https://doi.org/10.3390/ijms25010456
Ruzzi, F	Ruzzi, F.; Semprini, M.S.; Scalambra, L.; Palladini, A.; Angelicola, S.; Cappello, C.; Pittino, O.M.; Nanni, P.; Lollini, P.-L. Virus-like Particle (VLP) Vaccines for Cancer Immunotherapy. *Int. J. Mol. Sci.* **2023**, *24*, 12963. https://doi.org/10.3390/ijms241612963
Khunti, N	Khunti, N.; Kumar, M.; Datta, M.; Harelimana, J.d.D.; Harms, M.; Albers, D.; Kirchhoff, F.; Münch, J.; Stenger, S.; Buske, C.; et al. CXCR4 Inhibition Enhances the Efficacy of CD19 Monoclonal Antibody-Mediated Extermination of B-Cell Lymphoma. *Int. J. Mol. Sci.* **2025**, *26*, 2024. https://doi.org/10.3390/ijms26052024
Bloom, M	Bloom, M.; Podder, S.; Dang, H.; Lin, D. Advances in Immunotherapy in Hepatocellular Carcinoma. *Int. J. Mol. Sci.* **2025**, *26*, 1936. https://doi.org/10.3390/ijms26051936
Trocchia, M	Trocchia, M.; Ventrici, A.; Modestino, L.; Cristinziano, L.; Ferrara, A.L.; Palestra, F.; Loffredo, S.; Capone, M.; Madonna, G.; Romanelli, M.; et al. Innate Immune Cells in Melanoma: Implications for Immunotherapy. *Int. J. Mol. Sci.* **2024**, *25*, 8523. https://doi.org/10.3390/ijms25158523
Dalens, L	Dalens, L.; Lecuelle, J.; Favier, L.; Fraisse, C.; Lagrange, A.; Kaderbhai, C.; Boidot, R.; Chevrier, S.; Mananet, H.; Derangère, V.; et al. Exome-Based Genomic Markers Could Improve Prediction of Checkpoint Inhibitor Efficacy Independently of Tumor Type. *Int. J. Mol. Sci.* **2023**, *24*, 7592. https://doi.org/10.3390/ijms24087592
